# Book Reviews

**Published:** 1897-06

**Authors:** 


### BOOK REVIEWS.

---

DISEASES OF THE DOG AND THEIR TREATMENT. By PROFESSOR GEORG MÜLLER, Professor at the Veterinary School of Dresden. Translated, revised, and augmented by ALEXANDER GLASS, A.M., V.S., Lecturer on Canine Pathology at the University of Pennsylvania. With ninety-three illustrations. Published by W. Horace Hoskins, Philadelphia, 1897.

THE translation of Professor Müller's exhaustive work on *Die Krankheiten des Hundes* marks the beginning of a new era in canine pathology for English-speaking veterinarians. Heretofore there has been a great dearth of reliable information upon this subject. It is true that a number of books upon diseases of the dog have been published, notably those of Hill, Steel, and Mills; but these works are not of a thoroughly scientific character. They are written for the layman as well as the veterinarian, and are little more than compilations of the popular knowledge of these matters. They do not consider the science of the subjects of which they treat in the thoroughgoing, essential way that the diseases of other animals have been considered. The work translated by Dr. Glass is in all respects a scientific work, and while it will, no doubt, be of much value to many intelligent laymen, it is not written for them, but it is purely a professional work, and is based upon the best and most recent developments of modern physiology, pathology, bacteriology, and therapeutics, supplemented by the extensive practical experience of the author and translator.

The book differs so materially from anything that has previously appeared that it seems well briefly to review some of its excellent and unique features. The first chapter is on General Examination, and in the short space of ten pages describes the clinical significance of the general symptoms that dogs present when suffering from various diseases. The following chapters are on diseases of the various systems and organs, the infectious and constitutional diseases and surgical conditions. Each chapter is preceded by a brief general review of the points to be considered in the examination of the organs or parts of the body discussed. In many cases, as in the chapter upon Diseases of the Digestive Tract, Diseases of the Respiratory Organs, Diseases of the Nervous System, Diseases of the Circulatory Apparatus, etc., the preliminary discussion includes the topographical anatomy illustrated by very clear and instructive diagrams and pictures.

The methods to be employed in examining the various parts of the body are also considered in great detail, and include the use of various instruments, microscopic, physical, and chemical tests. Each disease is considered minutely in respect to its general characteristics, its etiology, pathological anatomy, clinical symptoms, diagnosis, prognosis, and treatment. Under the first heading the distribution, general prevalence, and importance of the disease are spoken of. The etiology is considered in the light of modern medicine, and numerous valuable points are brought out in this connection that have not previously appeared in English writings. The pathological anatomy is of the most practical character, and every point that is mentioned can be applied to advantage by the clinician. The clinical symptoms are considered in such great detail that it is evident that the author is not only a careful clinician and pathologist, but a comparative psychologist as well. The treatments recommended are in no case of the indefinite "shotgun" variety, but are all carefully considered and based upon an accurate knowledge of the nature of the disease and the actions of the various drugs employed. Much stress is laid upon hygienic and dietetic treatment. The chapter on Diseases of the Bones and Articulations is especially complete and valuable, and contains a great deal of new material. To illustrate the thorough manner in which these subjects are considered a mere catalogue of the subdivisions of this chapter is here reproduced: rhachitis, diseases of the joints, inflammation of the joints, acute synovial inflammation of the joints, chronic synovial inflammation of the joints, purulent inflammation of the joints, rheumatic inflammation of the joints, disease-producing malformation of the joints, injuries to the joints, wounds of the joints, contusions of the joints, distortions of the joints, luxation of the joints, dislocation of the lower jaw, dislocation of the elbow, dislocation of the patella, diseases of the bursa mucosa, muscular rheumatism, fractures of bones, amputation and exarticulation of bones.

The chapter on Hernia is divided as follows: description of hernia, reducible hernia, irreducible hernia, inguinal hernia, method of castration, sarcocele, hydrocele, umbilical hernia, femoral hernia, perineal hernia.

The chapter on Diseases of the Eyes is divided as follows: affections of the eyelids, closure of the eyelids, entropion, ectropion, diseases of the conjunctiva, inflammation of the conjunctiva, catarrhal ophthalmia, purulent ophthalmia, diseases of the sclerotic coat of the eye, inflammation of the sclerotic coat, keratitis superficialis, keratitis profunda or parenchymatosa, abscess of the sclerotic membrane, ulceration of the sclerotic membrane, dermoid of the cornea, pterygium, injuries to the cornea, diseases of the lens, cataract, diseases of the sclerotic membrane, of the nervous portion of the eye, and of the vitreous humor, inflammation of the iris, purulent inflammation of the eye, dropsy of the anterior chamber (glaucoma), diseases of the optic nerve and retina, prolapsus of the eyeball.

The Diseases of the Skin are discussed in a very thorough fashion; particular attention is given to those caused by animal parasites.

The book is characterized throughout by the absence of speculation in reference to unproven theories and the practical application of all that is known in relation to the subjects of which it treats. Many formulæ are printed which have been used by the author in his work as director of the clinic for small animals at the Dresden Veterinary School, and by the translator in connection with his duties at the Veterinary Department of the University of Pennsylvania and in his extensive canine practice.

The publisher is to be congratulated upon the excellent appearance of the work that marks his entrance into this new field. It is well known to the veterinary profession that it is his habit to do with great thoroughness everything that he undertakes, and they will, therefore, not be surprised to find this book as engaging in its general appearance as could be produced by any publisher who had devoted his life to the business. It is but fitting that the advent of a work destined to remain an authority for years to come, and the inauguration of a new publisher of veterinary works, should occur simultaneously and under such auspicious circumstances. May both have the prosperity they so richly deserve.

LEONARD PEARSON.

---

HANDY MANUAL OF VETERINARY MEDICINE, now in process of preparation under direction of M. Stalker, V.S., Dean Veterinary Department, Iowa State College, by G. E. Nesom, B.Sc., and Dr. R. A. Craig. This reference manual is designed for veterinarians, students, stockmen, and farmers, and is to appear in a few weeks. It is to contain handy reference on anatomy, dentition, physiology, hygiene, diseases and remedies, classified dose-table, surgery, obstetrics, poisons and antidotes, government rules for stock and meat-inspection, practical stock-feeding, history of domestic animals, list of State colleges having courses in veterinary medicine, State laws regulating the inspection of animals affected with contagious diseases, and much other useful information.

---

THE well-known publishing house of W. R. Jenkins, New York City, announces the issuance of *A Text-book on Veterinary Ophthalmology*, by George G. Van Mater, M.D., D.V.S., Professor of Ophthalmology in the American Veterinary College; also the early publication of a work by W. E. A. Wyman, Professor at the Clemson Agricultural and Mechanical College, South Carolina, on the *Clinical Diagnosis of Lameness in Horses*; also *A Compend of Veterinary Obstetrics*, by W. H. Dalrymple, M.R.C.V.S., of New Orleans, La., late professor in the Department of Veterinary Science in the Louisiana State University and A. and M. College; also *Exercises on Veterinary Research*, by C. J. Cadiot, translated by A. Bitting, M.D.V.S., with notes by Professor A. Liautard; also another work on *Practical Toxicology*, for professors and students, by Professor Dr. Rudolph Cobert, translated and edited by L. H. Friedberg, of New York, Professor of Chemistry and Toxicology in the American Veterinary College.

*The Veterinary Blue Book.* Unavoidable circumstances have delayed the publication of the *Blue Book*, now in the hands of the printer, but it will be published in June. Names sent in the first week of June, if not in time for the *Blue Book*, will be in time for the addenda. Persons desiring their names inserted should send their address, telephone number, and office hours. For further information, address R. S. Huidekoper, No. 154 W. Fifty-seventh Street, New York City.

The publication of this book will be an innovation not before attempted in veterinary circles, and we hope it may ultimately lead to the publication of a directory of the entire veterinary profession in North America.

